# Validation of the German Version of the New Freezing of Gait Questionnaire for People with Parkinson‘s Disease

**DOI:** 10.1155/2021/8841679

**Published:** 2021-01-13

**Authors:** Jana Seuthe, Kristina Kuball, Ann-Kristin Hoffmann, Burkhard Weisser, Günther Deuschl, Christian Schlenstedt

**Affiliations:** ^1^Department of Neurology, University Hospital Schleswig-Holstein, Christian-Albrechts-University, Kiel 24105, Germany; ^2^Department of Sports Sciences, Christian-Albrechts-University, Kiel, Germany; ^3^Neurology Department, Seegeberger Kliniken, Bad Segeberg 23795, Germany

## Abstract

Freezing of gait (FOG) in Parkinson's disease (PD) is a highly disabling symptom which impacts quality of life. The New FOG Questionnaire (NFOG-Q) is the most commonly used tool worldwide to characterize FOG severity in PD. This study aims to provide a German translation of the NFOG-Q and to assess its validity in people with PD. The questionnaire was translated using forward-backward translation. Validity was tested in 57 PD patients with FOG via Cronbach's alpha for internal consistency and Spearman correlations with several clinical measures to quantify disease severity, mobility, fall risk, and cognitive state for convergent and divergent validity. The German version of the NFOG-Q shows good internal consistency (C*α* = 0.84). Furthermore, the NFOG-Q score was significantly correlated with the MDS-UPDRS III, H&Y stage, Timed Up and Go test, and the subjective fear of falling (FES-I). The lack of correlation with cognition (MoCA) points towards good divergent validity. This study provides a German version of the NFOG-Q which proved to be valid for the assessment of FOG severity in individuals with PD.

## 1. Introduction

Freezing of gait (FOG) is a debilitating symptom in people with Parkinson's disease (PD) and is defined as a “brief, episodic absence or marked reduction of forward progression of the feet despite the intention to walk” [1]. It is often associated with falls due to the abrupt disruption of gait and also negatively impacts quality of life [2]. Due to its unpredictable and paroxysmal nature, it is often difficult to assess [3]. FOG commonly occurs during movements such as gait initiation, turning, when navigating through a narrow space (e.g., doorway and cluttered area), when reaching a destination, or when presented with an additional cognitive or stress component during gait [4]. So far, no gold standard exists to diagnose FOG and to measure FOG severity. Different approaches have been used such as self-reported measures using questionnaires [5], FOG provoking gait trajectories [6], or instrumented analysis with the use of wearable sensors [7]. In clinical routine, there can be a high false negative rate of patients subjectively reporting FOG which is not visible during the visit and thus not measured objectively. Hence, standardized assessment tools are needed to detect FOG and quantify its frequency and severity in order to adjust therapy and develop therapeutic approaches. Despite the shortcomings of questionnaire tools, they can be helpful as they are easy and quick to conduct and they may provide useful information of experiences in daily-life activities of the patient. Therefore, the New Freezing of Gait-Questionnaire (NFOG-Q), an updated version the Freezing of Gait Questionnaire (FOG-Q) [8], had been developed [9]. The NFOG-Q consists of 9 items and can be administered in approx. 10 minutes. It covers multiple dimensions of the symptom. Not only does it quantify the frequency and the duration of FOG in various situations but it also investigates the psychological strain and the consequences for daily activities for the patient, which can be especially helpful for clinical decisions regarding treatment. So far, the NFOG-Q has been widely used as one of the most relevant measures to quantify the occurrence and the severity of FOG [10–13]. To the best of our knowledge, no validated German translation of the NFOG-Q exists.

The aim of this study is to develop a German translation of the NFOG-Q, which can be used consistently in German-speaking countries and to examine its validity for people with PD with FOG. Given the association of FOG to disease severity, balance, fall risk, and cognition, we suspect the German translation of the NFOG-Q to be related to several clinical measures representing these domains.

## 2. Methods

### 2.1. Subjects

Fifty-seven people with idiopathic PD and FOG were examined for this study. Subjects were recruited from the outpatient clinic, the neurological and neurogeriatric ward, at the University Hospital Schleswig-Holstein, Kiel, Germany (*n* = 39), and from Segeberger Kliniken, Bad Segeberg, Germany (*n* = 18). Inclusion criteria were the diagnosis of idiopathic PD (according to UK PD Brain Bank criteria), preserved walking ability, and patient-reported occurrence of FOG in the past 4 weeks, by asking “Did you experience ‘freezing episodes' over the past month?” Exclusion criteria were any other neurological disease or orthopedic conditions restricting gait. The study was conducted according to the ethical principles of the Declaration of Helsinki and was approved by the local ethical committee of the University Hospital Schleswig-Holstein. All participants provided written informed consent prior to participation.

### 2.2. Development of the German Version of the NFOG-Q

The NFOG-Q is a subjective patient-reported measure [9] with 9 items using 2- to 5-point ordinal scales. It consists of three parts. The first part (item 1) aims to differentiate between freezers and nonfreezers by asking about the occurrence of FOG within the past month. Furthermore, it offers a video with different examples of different types and severities of FOG to visualize the symptom for patients that might not know what FOG is or are unsure if what they are experiencing is FOG. The second part (items 2–6) characterizes the severity of FOG by asking how often the freezing occurs in specific situations and how long episodes last. The third part (items 7–9) investigates how FOG affects people with PD in their everyday life. According to the 8 items of part II and III the total score can range between 0 and 28 points, with higher scores representing more severe FOG.

The cross-cultural validation was carried out in two stages. First, a bilingual native German speaker, who was aware of the concepts being examined in the questionnaire, translated the original NFOG-Q from English into German (JS). After this translation, a retranslation was conducted by another bilingual native German speaker (CS), who was familiar with the field and with the original English NFOG-Q. Finally, the original version and the retranslated version were reviewed by the two translators for incongruencies, and differences were discussed with another movement disorders specialist. Necessary changes were made to ensure comprehensibility and clarity. The German version of the NFOG-Q can be downloaded as supplemental material online.

### 2.3. Testing Procedure and Assessment Tools

For this study, we applied a cross-sectional design. Tests were carried out during the ON state of medication, to ensure patients were in a good physical state regarding their motor and nonmotor symptoms. Participants were not assisted by caregivers during the assessment of the NFOG-Q. To assess validity of the German version of the NFOG-Q, the translated NFOG-Q was related to the following clinical measures: to assess overall disease severity, the Movement-Disorders Society-Unified Parkinson's Disease Rating Scale (MDS-UPDRS) part III and the Hoehn and Yahr (H&Y) scale was conducted. Balance and mobility were measured using the Timed Up and Go test (TUG). To assess the impact of cognition, the TUG was performed under single task (ST) and dual task (DT) condition (serial threes backward subtraction), and DT cost (difference between ST and DT) was calculated. Furthermore, the Montreal Cognitive Assessment (MoCA) was conducted. Fall risk was assessed using the Falls Efficacy Scale-International (FES-I) and number of falls over the past 6 months.

### 2.4. Statistical Analysis

For the analysis, descriptive statistics (mean, standard deviation, and minimum and maximum) was calculated to characterize the distribution or possible floor or ceiling effects of outcome measures. Internal consistency reliability was assessed using Cronbach's *α* (Ca). Recent literature highlights the problem with the arbitrary but often used value of 0.7 for good internal consistency [14], which might not always be sufficient to claim good internal consistency. That is why, we will not provide an interpretation of Ca by different cutoffs previously mentioned in the literature, but rather interpret the results in the context of the investigated instrument. Generally, a high Ca reflects high internal consistency [15]. Additionally, to investigate convergent and divergent validity, Spearman's rho correlations were calculated between the different clinical measures. Furthermore, a subanalysis was performed for participants with cognitive impairments (MoCA < 21) [16]. It is noteworthy that inconsistency exists with regard to cutoff values for cognitive impairments in PD [16].

Statistical analysis was performed using RStudio [17].

## 3. Results

No major differences were found between the retranslated English version of the NFOG-Q and the original version. Minor differences (slight variation in the phrasing of the questions) were discussed between the two translators and the third movement disorders specialist to get a final German version of the NFOG-Q to which all investigators agreed to.

Participant characteristics can be found in Table 1. Total scores of the German version of the NFOG-Q ranged between 7 and 28 with a mean of 17.82 (±6.29). Cronbach's *α* was 0.84 for the scale, suggesting a high internal consistency. The standardized Ca for each item can be found in Table 2.

The total NFOG-Q score of the German version was significantly correlated with the MDS-UPDRS III (*r* = 0.280, *p*=0.038) and with the H&Y scale (*r* = 0.390, *p*=0.003) (Table 3). Furthermore, it was correlated with the TUG (ST: *r* = 0.440, *p* < 0.001; DT: *r* = 0.535, *p* < 0.001; DT cost: *r* = 0.384, *p*=0.005) and the fear of falling assessed by the FES-I (*r* = 0.600, *p* < 0.001). We found no significant correlation of the total NFOG-Q score with the disease duration (*r* = −0.064, *p*=0.643), the retrospective number of falls (*r* = 0.179 *p*=0.183), and the MoCA (*r* = −0.148, *p*=0.272).

Excluding cognitively impaired participants revealed that Cronbacha's *α* only changed marginally to 0.83 (*n* = 45). However, correlation of the NFOG-Q score with the MDS-UPDRS-III in the subgroup without major cognitive impairment was not significant. For details of all correlations in this subanalysis, see Table 4. Internal consistency for individuals with major cognitive impairment (*n* = 12, MoCA < 21) was also high (Ca = 0.89).

## 4. Discussion

A German translation of the NFOG-Q was developed, and its validity was investigated in people with PD and FOG. We found high internal consistency for our German version of the NFOG-Q, as the items are interrelated but not redundant. Our results are in line with previous studies: internal consistency of the original version of the FOG-Q has been reported to range between 0.89 and 0.9 (Cronbach's *α*) for the original English version [5] and 0.83 for the German version [18]. Similarly, the NFOG-Q in the English version showed a Cronbach's *α* of 0.84 [9], comparable to what we found.

In contrast to the German validation of the FOG-Q [18], we found the total score to be significantly correlated with the MDS-UPDRS III, as well as with the TUG. This is the proof of good construct validity as general motor symptoms and gait function are thought to be related with FOG [19]. Furthermore, H&Y stage also showed a significant correlation with the NFOG-Q total score of our German version. It has been shown previously that the general occurrence of FOG [20] and also severity of FOG [21, 22] is linked to overall disease severity, which is in line with our findings.

As expected, individuals with higher NFOG-Q scores also presented with a higher fear of falling (FES-I), which was also found in previous cross-cultural validation studies of the FOG-Q [21, 23]. As FOG can disrupt gait abruptly, this FOG-related fear of falling and a generally large impact of FOG on their daily activities translates to a higher overall fear of falling.

Our German version of the NFOG-Q was associated with the performance of the TUG as previously reported for the FOG-Q [22, 23]. It was also correlated with the TUG while performing an additional cognitive task but not with the MoCA. These rather inconsistent results reflect current findings about the association between FOG and cognition as some studies support this association [24–26] whereas others do not [27]. The fact that the NFOG-Q was not correlated with the MoCA but with the dual-tasking TUG might reflect that FOG is less related to global cognition [27] than to specific cognitive impairments such as dual tasking. The impaired simultaneous conduction of a cognitive and motor task might represent increased cognitive control (due to reduced automaticity) in PD + FOG when performing motor tasks, reducing cognitive resources for the cognitive task.

Assessment of FOG through questionnaire tools relies on the subjective perception of each patient and how well they recall the incidences of the symptom. Previous research has shown that PD + FOG commonly underestimates the severity of their symptom [9]. Furthermore, the classification of freezer and nonfreezer can sometimes be problematic. It could be the case that OFF akinesia is mistaken for FOG by people with PD, but this has not been investigated yet. Moreover, it is noteworthy that the NFOG-Q should ideally only be administered with nondemented individuals [18]. This was not the case in this study, as some subjects scored below proposed cutoff values in the MoCA. As participants need to recall incidents of FOG retrospectively for a period of 4 weeks and need to have good self-perception, this adds some bias to the current work. However, results revealed similar Ca when excluding cognitively impaired participants and when analyzing the subgroup of cognitively impaired subjects separately.

The following limitations have to be noticed: first, there may be some bias in the translation process, as the retranslator was also familiar with the original English NFOG-Q. Second, no a priori sample size calculation has been performed. However, post hoc power analysis revealed a power of 0.7 for the correlation of the NFOG-Q with the MDS-UPDRS and above 0.9 for the other significant correlations.

Recent work has shown poor test-retest-reliability resulting in a high rate of minimal detectable change of the NFOG-Q [11]. This suggests that the NFOG-Q might be less useful to detect treatment effects. The use of instrument-based measures therefore might be a good addition when investigating FOG-based treatment interventions.

## 5. Conclusion

This study provides a German version of the NFOG-Q which proved to be valid for the assessment of FOG severity in people with PD. However, for the use in longitudinal studies, the additional use of more objective methods should be considered. Clinicians, therapists, and researchers can use the questionnaire to characterize the severity of the symptoms as well as consequences on daily life in their patients or study participants. Especially for clinical routine, it is a useful tool and quick to administer. The German version of the NFOG-Q can be used consistently in German-speaking countries and can be downloaded at (https://www.neurologie.uni-kiel.de/de/neuromechanik-neurorehabilitation/downloads/nfogq-german.pdf.

## Figures and Tables

**Table 1:** Participant characteristics (*n* = 57).

Outcome measure	Mean ± SD (range)
Age (y)	69.91 ± 9.882 (48–86)
Sex (m/f)	39/18
Disease duration (y; *n* = 55)	12,42 ± 6,863 (1–30)
H&Y (1/2/3/4) (*n* = 55)	1/6/36/13
MDS-UPDRS III	30.61 ± 16.12 (3–69)
TUG in sec (ST)	19.14 ± 16.45 (8–107)
TUG in sec (DT)	23.73 ± 19.89 (9–128)
FES-I	33.81 ± 11.53 (16–62)
Number of falls^a^	3.79 ± 6.57 (0–40)
MoCA	23.65 ± 3.633 (15–29)

^a^In the past 6 months. SD = standard deviation, y = years, m = male, f = female, H&Y = Hoehn and Yahr stage, MDS-UPDRS-III = Movement Disorder Society-Unified Parkinson's Disease Rating Scale part III, TUG = Timed Up and Go, ST = single task, DT = dual task, FES-I = Falls Efficacy Scale-International, MoCA = Montreal Cognitive Assessment.

**Table 2:** Standardized Cronbach's *α* for each ordinal-scale item.

NFOG-Q item	Cronbach's *α*
2	0.84
3	0.83
4	0.81
5	0.82
6	0.81
7	0.82
8	0.83
9	0.82

**Table 3:** Correlations of NFOG-Q with other outcome measures (*n* = 57).

Outcome measure	Spearman's rho	*p* value
MDS-UPDRS-III	0.280	0.035^*∗*^
H&Y	0.390	0.003^*∗*^
Disease duration	−0.074	0.594
TUG (ST)	0.425	<0.001^*∗∗*^
TUG (DT)	0.525	<0.001^*∗∗*^
DT cost	0.384	0.005^*∗*^
FES-I	0.583	<0.001^*∗∗*^
Number of falls^a^	0.175	0.192
MoCA	−0.155	0.250

^a^In the past 6 months, MDS-UPDRS-III = Movement Disorder Society-Unified Parkinson's Disease Rating Scale part III, H&Y = Hoehn and Yahr stage, TUG = Timed Up and Go, ST = single task, DT = dual task, FES-I = Falls Efficacy Scale-International, MoCA = Montreal Cognitive Assessment, ^*∗*^significant, ^*∗∗*^highly significant.

**Table 4:** Correlations of NFOG-Q with other outcome measures only for subjects without major cognitive impairment (*n* = 45).

Outcome measure	Spearman's rho	*p* value
MDS-UPDRS-III	0.266	0.085
H&Y	0.425	0.005^*∗*^
Disease duration	0.024	0.881
TUG (ST)	0.493	<0.001^*∗∗*^
TUG (DT)	0.567	<0.001^*∗∗*^
DT cost	0.433	0.006^*∗*^
FES-I	0.595	<0.001^*∗∗*^
Number of falls^a^	0.120	0.445
MoCA	−0.074	0.638

^a^In the past 6 months, MDS-UPDRS-III = Movement Disorder Society-Unified Parkinson's Disease Rating Scale part III, H&Y = Hoehn and Yahr stage, TUG = Timed Up and Go, ST = single task, DT = dual task, FES-I = Falls Efficacy Scale-International, MoCA = Montreal Cognitive Assessment, ^*∗*^significant, ^*∗∗*^highly significant.

## Data Availability

The clinical data used to support the findings of this study have not been made publicly available due to restrictions by the local ethics committee. Data can be shared upon request with researchers from the field with approval of the ethical committee in charge.
